# A polyphasic taxonomy analysis reveals the presence of an ecotype of *Rahnella contaminans* associated with the gut of *Dendroctonus*-bark beetles

**DOI:** 10.3389/fmicb.2023.1171164

**Published:** 2023-04-27

**Authors:** Flor N. Rivera-Orduña, Rosa María Pineda-Mendoza, Brenda Vega-Correa, María Fernanda López, Claudia Cano-Ramírez, Xiao Xia Zhang, Wen Feng Chen, Gerardo Zúñiga

**Affiliations:** ^1^Departamento de Microbiología, Escuela Nacional de Ciencias Biológicas, Instituto Politécnico Nacional, Mexico City, Mexico; ^2^Departamento de Zoología, Escuela Nacional de Ciencias Biológicas, Instituto Politécnico Nacional, Mexico City, Mexico; ^3^Institute of Agricultural Resources and Regional Planning, Chinese Academy of Agricultural Sciences, Beijing, China; ^4^State Key Laboratory for Agro-Biotechnology and Ministry of Agriculture Key Lab of Soil Microbiology, College of Biological Sciences, China Agricultural University, Beijing, China

**Keywords:** Enterobacteriaceae, gut symbiont, bark beetles, *Dendroctonus*, gut bacteriome

## Abstract

Species belonging to the genus *Rahnella* are dominant members of the core gut bacteriome of *Dendroctonus*-bark beetles, a group of insects that includes the most destructive agents of pine forest in North and Central America, and Eurasia. From 300 isolates recovered from the gut of these beetles, 10 were selected to describe an ecotype of *Rahnella contaminans*. The polyphasic approach conducted with these isolates included phenotypic characteristics, fatty acid analysis, 16S rRNA gene, multilocus sequence analyses (*gyrB*, *rpoB*, *infB,* and *atpD* genes), and complete genome sequencing of two isolates, ChDrAdgB13 and JaDmexAd06, representative of the studied set. Phenotypic characterization, chemotaxonomic analysis, phylogenetic analyses of the 16S rRNA gene, and multilocus sequence analysis showed that these isolates belonged to *Rahnella contaminans*. The G + C content of the genome of ChDrAdgB13 (52.8%) and JaDmexAd06 (52.9%) was similar to those from other *Rahnella* species. The ANI between ChdrAdgB13 and JaDmexAd06 and *Rahnella* species including *R. contaminans*, varied from 84.02 to 99.18%. The phylogenomic analysis showed that both strains integrated a consistent and well-defined cluster, together with *R. contaminans*. A noteworthy observation is the presence of peritrichous flagella and fimbriae in the strains ChDrAdgB13 and JaDmexAd06. The *in silico* analysis of genes encoding the flagellar system of these strains and *Rahnella* species showed the presence of *flag*-1 primary system encoding peritrichous flagella, as well as fimbriae genes from the families type 1, α, β and σ mainly encoding chaperone/usher fimbriae and other uncharacterized families. All this evidence indicates that isolates from the gut of *Dendroctonus*-bark beetles are an ecotype of *R. contaminans*, which is dominant and persistent in all developmental stages of these bark beetles and one of the main members of their core gut bacteriome.

## Introduction

The genus *Rahnella* belongs to the family *Yersiniaceae* and comprises 14 taxonomically valid species^1^: *Rahnella aquatilis* (Izard et al., 1979), *R*. *bruchi, R*. *inusitata*, *R*. *victoriana*, *R*. *variigena*, *R*. *woolbedingensis* (Brady et al., 2014), *R*. *aceris* (Lee et al., 2020), *R. contaminans, R*. *laticis* (Jeon et al., 2021), *R*. *bonaserana, R*. *ecdela, R*. *perminowiae*, *R*. *rivi* (Brady et al., 2022), and *R*. *sikkimica* (Kumar et al., 2022). *Rahnella* species reside in a broad range of environments such as soil (Kim et al., 1997), seeds (Kämpfer, 2005), phloem and bark (Gonzalez-Escobedo et al., 2018), roots (Izumi et al., 2006), water (Izard et al., 1979), food (Kämpfer, 2005), human clinical samples (Alballaa et al., 1992), and extremophile environments (Mukhia et al., 2022). In addition, several studies have revealed the presence of this genus in association with insects such as bark beetles (Briones-Roblero et al., 2017a; Fabryová et al., 2018), cockroaches (Akbari et al., 2014), honeybees (Kačániová et al., 2020), and mosquitoes (Charan et al., 2013).

The bark beetles *Dendroctonus* spp. (Curculionidae: Scolytinae) are phloeophagous insects that spend most of their life cycle under the tree’s bark and are one of the most destructive pine forest agents in North and Central America, and Eurasia (Six and Bracewell, 2015). *Rahnella* strains have been isolated from the exoskeleton, galleries, and guts of these and other bark beetles (Morales-Jiménez et al., 2013; Durand et al., 2015; Mason et al., 2015; Hernández-García et al., 2017; Briones-Roblero et al., 2017a,b; Fabryová et al., 2018; Chakraborty et al., 2020). *Rahnella* is a member of the core gut bacteriome of *Dendroctonus* spp. (Hernández-García et al., 2017), and it has been persistently reported in all developmental stages of *Dendroctonus rhizophagus* (Briones-Roblero et al., 2017a). Furthermore, it has been shown that *Rahnella* strains from bark beetles play a fundamental role in the digestive and detoxification processes, since they are capable of degrading different substrates such as esters, lipids, and xylan (Briones-Roblero et al., 2017b; Pineda-Mendoza et al., 2022), recycling uric acid (Morales-Jiménez et al., 2013), and degrading or transforming different monoterpenes of the host trees of these insects (Boone et al., 2013; Xu et al., 2015, 2016).

In this study, *Rahnella* isolates associated with the gut of *Dendroctonus*-bark beetles were characterized following a polyphasic approach based on genotypic, phenotypic, genomic, and fatty acid assays. Our results show that these strains are an ecotype of the nominal species *Rahnella contaminans*, whose capabilities and ecological role in benefit of these insects are being explored.

## Materials and methods

### Isolation and preliminary molecular identification

Bacteria were isolated from 10 homogenized gut sets from larvae, pupae and adults of some *Dendroctonus* species collected in different geographical sites in Mexico. Prior to dissection, insects were superficially disinfected according to Briones-Roblero et al. (2017a). Insects were grouped and processed by location; thus, individuals of the same species from different locations were not mixed. Guts were homogenized with sterile polyethylene pestles into Eppendorf tubes containing phosphate-buffered saline (PBS) solution. Thereafter, serially diluted solutions were prepared and spread onto tryptic soy agar (TSA; 15 g L^−1^ of tryptone, 5 g L^−1^ of soytone, 5 g L^−1^ of sodium chloride, and 15 g L^−1^ of agar; BD Difco, United States) plates, which were incubated at 28°C for 48 h. Individual colonies were subsequently streaked on TSA plates and incubated under previous conditions. A total of 500 isolates were recovered from the gut of *Dendroctonus* species, which were stored in a sterile 30% (v/v) glycerol solution at −70°C.

Bacterial DNA was extracted from axenic colonies following the method reported by Hoffman and Winston (1987). Amplifications of the 16S rRNA gene were performed according to Briones-Roblero et al. (2017b). Amplicons were purified with GeneJET PCR Purification Kit (Thermo Fisher Scientific, Waltham, MA), and then sequenced in Macrogen Inc. (Seoul, Korea). All sequences were compared with sequences deposited in GenBank and EzBioCloud^2^ databases. A total of 300 sequences from our isolates matched with those from *Rahnella* species. These sequences and other phylogenetically closely related species were aligned using Clustal X v.2.1 (Larkin et al., 2007). A phylogenetic characterization based on the 16S rRNA gene was done using maximum likelihood in PhyML.^3^ The best nucleotide substitution model was determined using jModelTest v.2.1.7 (Darriba et al., 2012) under the Akaike information criterion. The nodes’ reliability was evaluated using a bootstrap test after 1,000 pseudoreplications.

**Figure 1:**
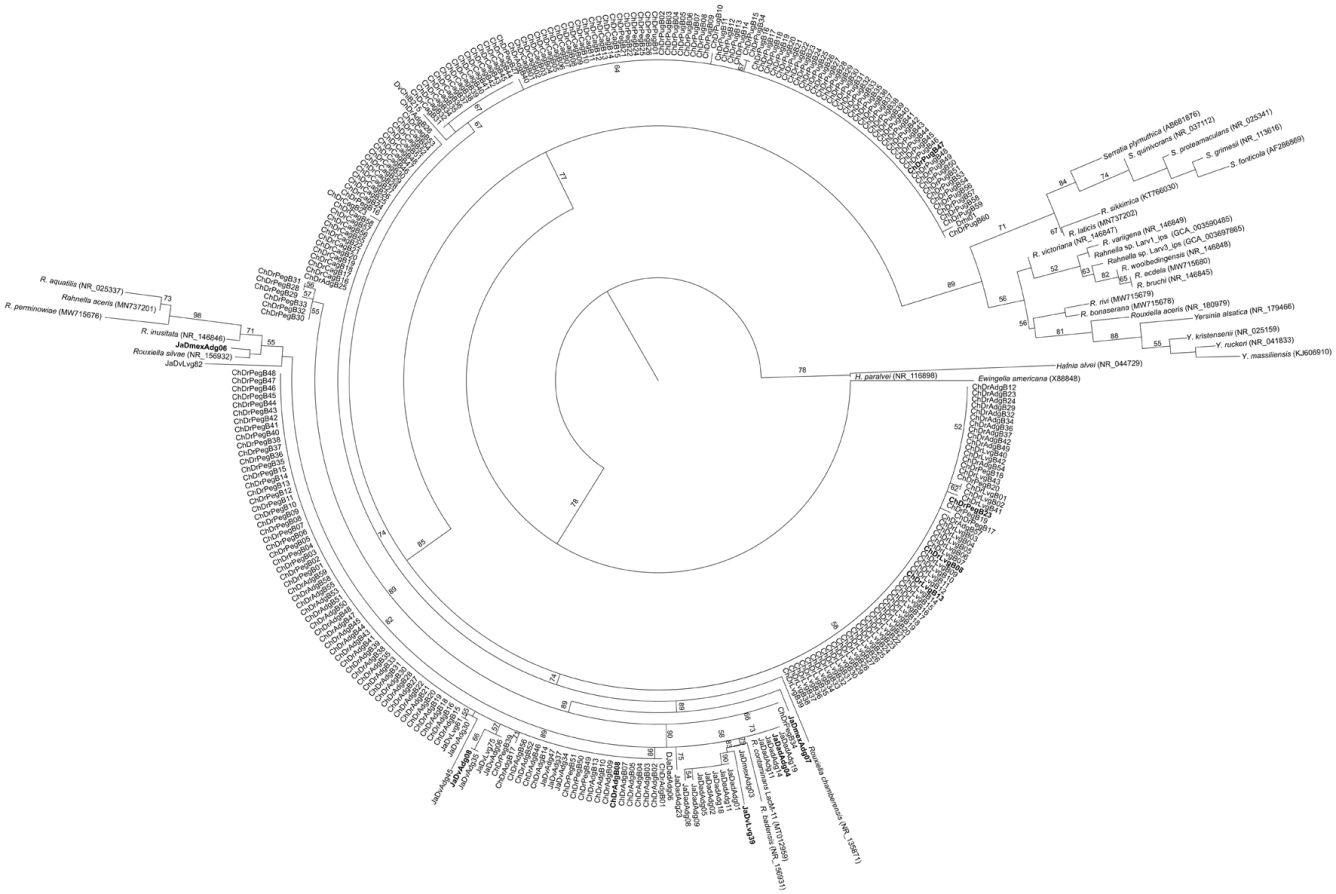
Maximum likelihood phylogeny of the 16S rRNA sequences of 300 *Rahnella* isolates and type strains of the genus *Rahnella.* The nucleotide substitution model used was GTR with a gamma parameter of 0.693. Bootstrap values >50% are shown at nodes. In bold are the sequences selected for other analyses in this study.

Based on the 16S rRNA gene phylogeny of 300 isolates (Figure 1), 10 *Rahnella* strains were selected for their physiological and molecular characterization (Table 1). The strains ChDrAdgB13 (accession number CDBB-2057 = NRRL B-65604) and JaDmexAd06 were selected for genome sequencing and observation of morphological characters.

**Table 1:** Bacterial isolates used in this study, *Dendroctonus* species, localities, and geographic references where they were collected.

Strain	Insect stage	Sources	Localities	Latitude	Longitude
ChDrAdgB13^T^	Adults	*Dendroctonus rhizophagus*	San Juanito, Bocoyna Municipality, Chihuahua	27° 45′	107° 38′
ChDrLvgB08	Larvae	*Dendroctonus rhizophagus*	San Juanito, Bocoyna Municipality, Chihuahua	27° 45′	107° 38′
ChDrPugB47	Pupae	*Dendroctonus rhizophagus*	San Juanito, Bocoyna Municipality, Chihuahua	27° 45′	107° 38′
ChDrPegB23	Pre-emerged adults	*Dendroctonus rhizophagus*	San Juanito, Bocoyna Municipality, Chihuahua	27° 45′	107° 38′
JaDmexAdg06	Adults	*Dendroctonus mexicanus*	Los Bancos, Pueblo Nuevo municipality, Durango	23° 39´	105° 44´
JaDmexAdg07	Adults	*Dendroctonus mexicanus*	Los Bancos, Pueblo Nuevo municipality, Durango	23° 39´	105° 44´
JaDadAdg04	Adults	*Dendroctonus adjunctus*	Nevado de Colima, Jalisco	19° 35´	103° 36´
JaDadAdg19	Adults	*Dendroctonus adjunctus*	Nevado de Colima, Jalisco	19° 35´	103° 36´
JaDvAdg08	Adults	*Dendroctonus valens*	Las Güilotas, Gomez Farias municipality, Jalisco	19° 51´	103° 23´
JaDvLvg39	Larvae	*Dendroctonus valens*	Las Güilotas, Gomez Farias municipality, Jalisco	19° 51´	103° 23´

### Phenotypic characterization

Ten isolates were cultured on TSA at 28°C for 48 h. Cellular morphology was observed in detail by transmission electron microscopy (JEOL JEM-2100) after negative staining with phosphotungstic acid hydrate (Sigma-Aldrich, United States). Gram staining was performed using the Gram stain kit (bioMérieux, Inc., United States) according to the manufacturer’s instructions. Oxidase and catalase activities were determined with N,N-dimethyl-p-phenylenediamine dihydrochloride and 3% (v/v) H_2_O_2_ solutions, respectively. Strains were cultured on TSA plates at 4, 10, 20, 28, 37, and 44°C to determine the growth temperature range for 48 h. Tryptic soy broth (TSB, Difco) medium was used to test the growth capability of the strains at different pH values. The medium was adjusted to a final pH of 4–10 using NaOH and HCI 1.0 M. Cultures were incubated at 28°C for up to 48 h. Growth was determined by measuring the absorbance at 600 nm using a Multiskan^™^ GO Microplate Spectrophotometer (Thermo Fisher Scientific, Waltham, MA, United States). The physiological and biochemical capacities of the strains were evaluated visually using the API 50CH and API 20E systems (bioMérieux Inc., United States) and Biolog GN2 Microplates (Biolog, Inc., United States) following the manufacturers’ instructions, and adjusting the inoculum to 95% T in GN/GP Inoculating Fluid (Biolog, Inc., United States) and incubated at 28°C for 24–48 h.

### Chemotaxonomic analysis

For the determination of cellular fatty acids, the isolates were grown in TSA at 28°C for 24 h. The extraction and methylation of fatty acids were performed according to the standard protocol of the Sherlock Microbial Identification System (MIS) (Sasser, 1990), and later identified and quantified using MIDI System software (MIDI Inc., United States).

## Genotypic characterization

### 16S rRNA gene and multilocus sequence analysis (MLSA)

The bacterial DNA of 10 isolates was extracted from axenic colonies following the method reported by Hoffman and Winston (1987). Phylogenetic inference analysis of these isolates was carried out as described above, including all nominal species of *Rahnella* and some species of the family *Yersiniaceae*. The 16S rRNA gene sequence pairwise similarity among these taxa was estimated in MatGAT v.2.01 (Campanella et al., 2003). In contrast, the amplification and partial sequencing of *gyrB*, *rpoB*, *infB*, and *atpD* housekeeping genes (MLSA) were carried out according to Brady et al. (2008, 2014). PCR products were purified with a GeneJET PCR Purification Kit (Thermo Scientific, Waltham, MA) according to the manufacturer’s protocol and sequenced in Macrogen Inc. (Seoul, Korea). All sequences were compared to those available from the GenBank database.^4^ For phylogenetic analysis, sequences of the housekeeping genes, including sequences of phylogenetically close bacteria species, were aligned independently in Clustal X v.2.1 and edited in Seaview v.5.0.4 (Gouy et al., 2010). Sequences of these genes were concatenated to perform a maximum likelihood phylogenetic analysis in PhyML.^5^ The best nucleotide substitution model for concatenated sequences was determined using jModelTest v.2.1.7 under the Akaike information criterion. The nodes’ reliability was evaluated by means of a bootstrap test after 1,000 pseudoreplications. Housekeeping gene sequences were deposited in the GenBank database under the accession number (MW648385–MW648424).

### Genome analysis

The isolatesxs ChDrAdgB13 and JaDmexAd06 were grown overnight in 10 ml of TSA broth at 28°C, with orbital shaking at 150 rpm. The genomic DNA was isolated using DNeasy^®^ Blood and Tissue kit according to the manufacturer’s protocol (QIAGEN, United States). The whole-genome sequencing was performed on the HiSeq2000 Illumina platform by Otogenetics Corporation (Norcross, GA, United States). The paired-end data were quality checked using FastQC v.0.11.3 (Babraham Institute, Cambridge, United Kingdom). Reads were filtered using a sliding window quality cutoff of Q4:15 in Trimmomatic v.036 (Bolger et al., 2014), and then high-quality reads were *de novo* assembled using Velvet v.1.2.10 (Zerbino and Birney, 2008). Genome completeness and potential contamination were estimated using RAST server v.2.0^6^ and KBase,^7^ respectively. Protein, rRNA, and tRNA-coding genes were identified using Prokka v.2.8^8^ (Seemann, 2014) and the Kyoto Encyclopedia of Genes and Genomes (KEGG) Automatic Annotation Server (KAAS).^9^ The genome sequences were submitted to GenBank under the BioProject number JAEMGT000000000 for ChDrAdgB13 and JARBHC000000000 for JaDmexAd06.

The G + C content of DNA was determined directly from the complete genome. The average nucleotide identity (ANI) and digital DNA–DNA hybridization (dDDH), using formula *d_4_* among genomes of two strains and *Rahnella* species, were estimated with the ANI Calculator at Kostas lab^10^ and GGDC 2.1,^11^ using the recommended settings in both servers, respectively.

### Phylogenomic analysis

Genome-based phylogeny from the isolates ChDrAdgB13, JaDmexAd06, nominal species of *Rahnella*, and other related species of *Yersiniaceae* was performed using the Type Strain Genome Server (TYGS)^12^ (Meier-Kolthoff and Göker, 2019). The phylogenomic tree was inferred using FastME^13^ (Lefort et al., 2015) and Genomic BLAST Distance Phylogeny (GBDP), which provides pairwise intergenomic distances generated using algorithm “trimming” and the distance formula *d_4_* (Meier-Kolthoff et al., 2013). The robustness of the relationships was evaluated with a bootstrap test after 100 pseudoreplicates. The tree was visualized using FigTree v.1.4.4 (Rambaut, 2012).

### Flagellar characterization

Given that there is a discrepancy between the type of flagella, peritrichous, or amphitrichous (polar), presented by the species of the genus *Rahnella* (Jeon et al., 2021; Brady et al., 2022), observations by TEM of isolates ChDrAdgB13 and JaDmexAd06 were performed to clarify the flagella arrangement of these isolates. In addition, based on the genomes of the 14 taxonomically valid species of *Rahnella*, we performed a comprehensive *in silico* analysis of genes encoding flagella. The annotation was performed using the Bacterial and Viral Bioinformatics Resource Center (BV-BRC) v.3.28.9^14^ and RAST server.

## Results

### Phenotypic features

**Figure 2:**
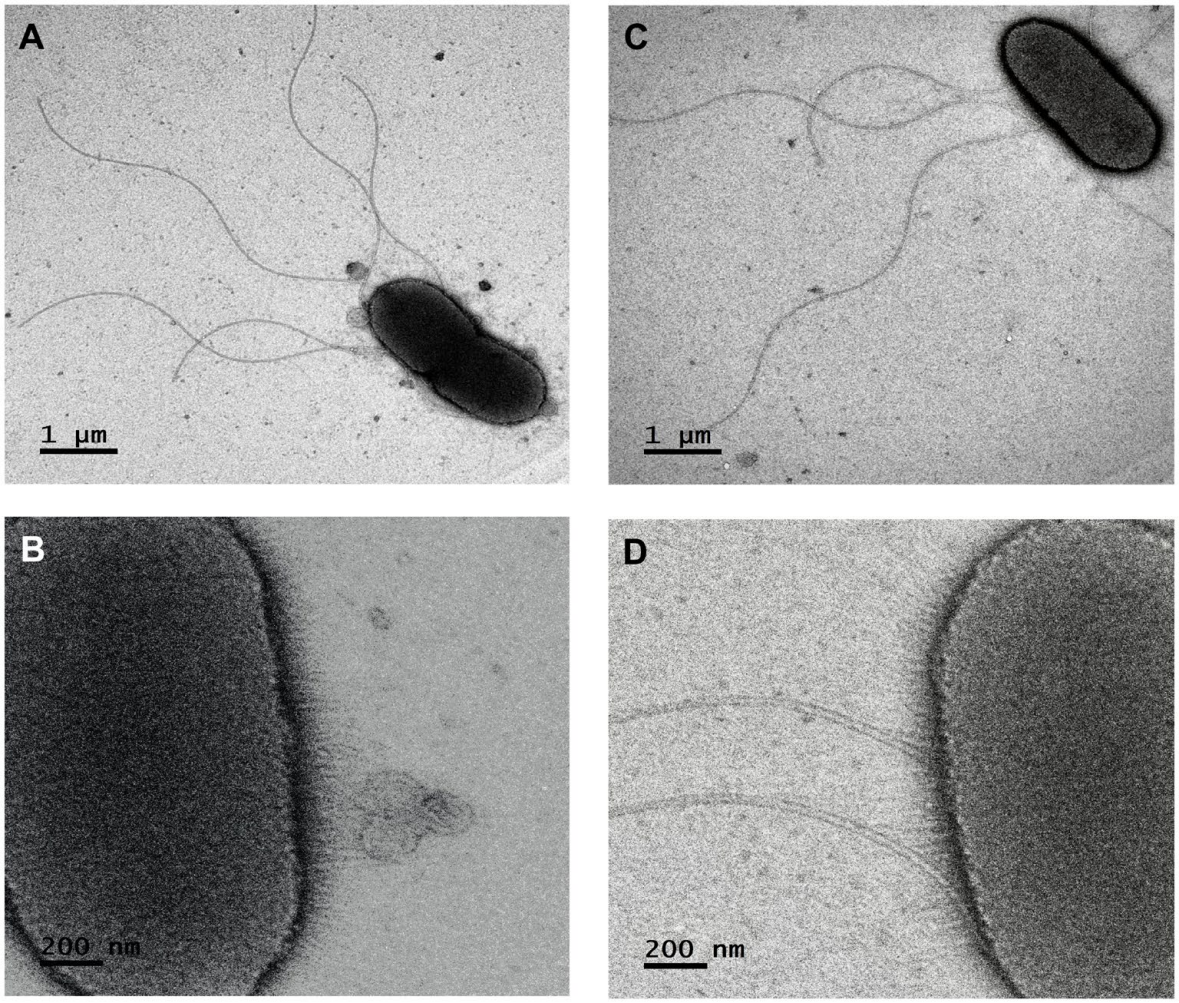
Scanning electron micrograph of the *Rahnella contaminans* ecotype. **(A,B)** flagella and fimbriae of strain ChDrAdgB13, and **(C,D)** of strain JaDmexAd06 (4,000×).

The colonies were white, round, and convex with the entire margin being smooth and bright. They did not produce a diffusible pigment on the TSA medium after incubation at 28°C for 48 h. The cells were Gram-negative rods (0.4–0.6 × 2–3.5 μm), motile with fimbriae, and peritrichous flagella (Figure 2). The growth occurred in a temperature range of 20–28°C and a pH range from 4 to 10, with an optimum temperature of 28°C and a pH 7.

The phenotypic characteristics of 10 isolates were similar in acid production and enzyme activities, but different in the carbon sources assimilated (Supplementary Table S1). Comparison of the phenotypic characteristics of the strains ChDrAdgB13 and JaDmexAd06, and type strains of *Rahnella* species were different in acid production and carbon sources assimilated, but similar in the enzyme activities except in the activity tryptophan deaminase (TDA) (Supplementary Table S1).

### Chemotaxonomic analysis

The cellular fatty acids of the 10 isolates were similar to those of the type strains of the *Rahnella* genus. The main fatty acid components of 10 isolates were similar among them [C_12:0_ (2.8–2.9%), C_14:0_ (5.5–5.6%), C_16:0_ (26.4–26.6%), C_17:0_ cyclo (22.7%), iso-C_16:1_, and/or C_14:0_ 3-OH (8.3–8.4% summed feature 2), and C_16:1_ ω7*c* and/or C_16:1_ω6c (15.4–15.5%, summed feature 3)] (Supplementary Table S2). Unfortunately, this comparison was not possible to perform with *R*. *contaminans* and *R*. *laticis* because both species were grown under anoxic conditions.

### Molecular characterization

The phylogenetic inference analysis with the 16S rRNA gene showed that the 10 selected isolates are distributed in a consistent clade (bootstrap value >70%) with species of *Rouxiella* and *Rahnella* including *R*. *contaminans*, *R*. *inusitata*, *R. aceris*, *R*. *aquatilis*, *R*. *perminowiae*, and some *Rouxiella* species. The rest of *Rahnella* type species formed another clade separate from it (Supplementary Figure S1). Pairwise similarity values were higher among 10 isolates and *R*. *contaminans* (≥99.92%) than between the isolates and type strains of *Rahnella* species (97.32–99.75%) (Supplementary Table S3).

**Figure 3:**
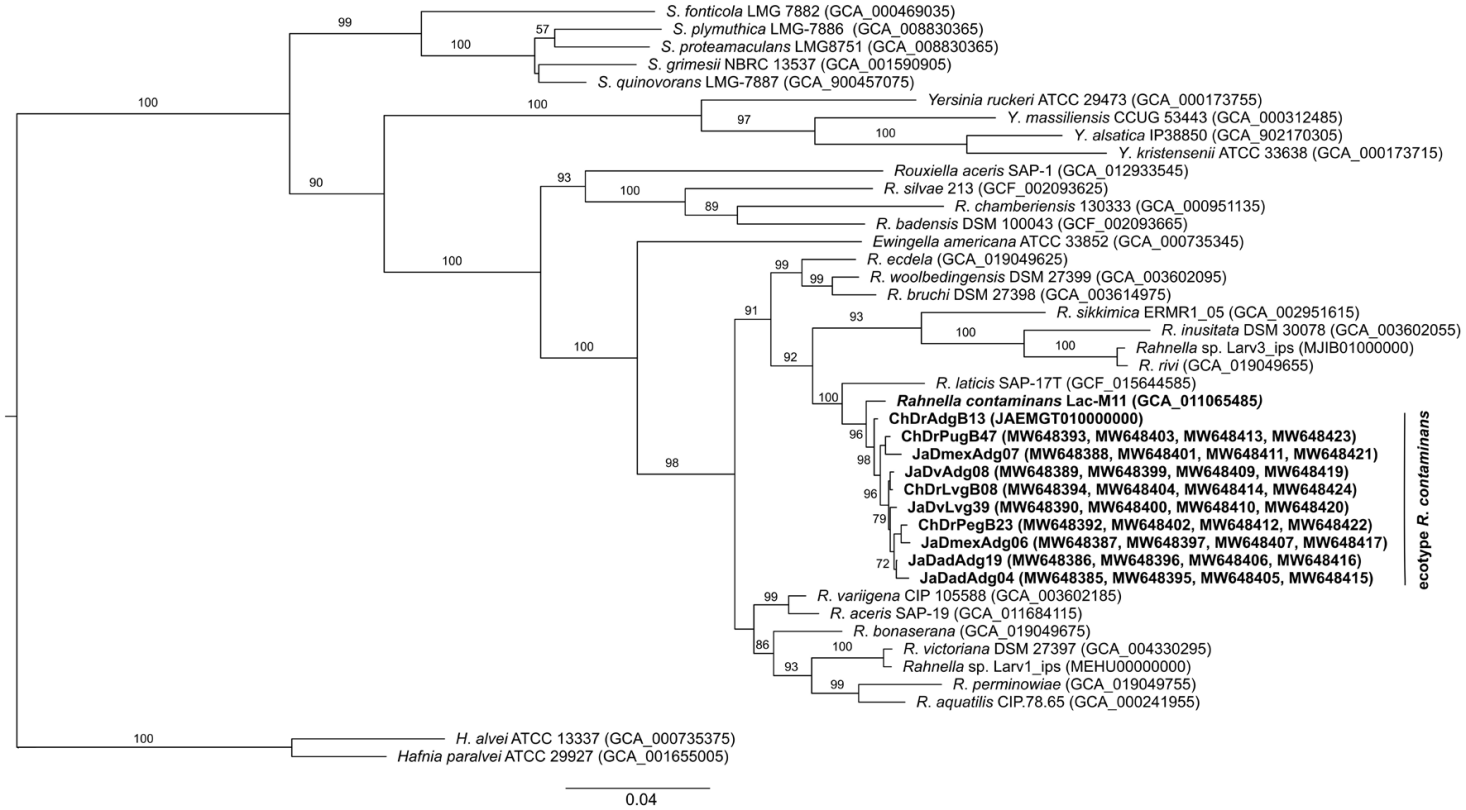
Maximum likelihood phylogenetic tree based on concatenated *gyrB*, *rpoB*, *infB*, and *atpD* gene sequences of 10 isolates and the type strains of the *Rahnella* species. The nucleotide substitution model was GTR + R according to Akaike criterion. Bootstrap values >50% after 1,000 pseudoreplicates are shown at nodes.

The maximum likelihood phylogenetic tree inferred using concatenated housekeeping genes showed that the 10 selected isolates and *R*. *contaminans* formed an independent cluster (bootstrap value >97%) close to *R*. *laticis* within the genus clade (Figure 3). Pairwise similarity values of concatenated housekeeping genes were higher, also among the 10 isolates and *R*. *contaminans* (98.98–99.67%), than between isolates and other *Rahnella* species (91.20–98.88%) (Supplementary Table S3).

### Genomic features

**Figure 4:**
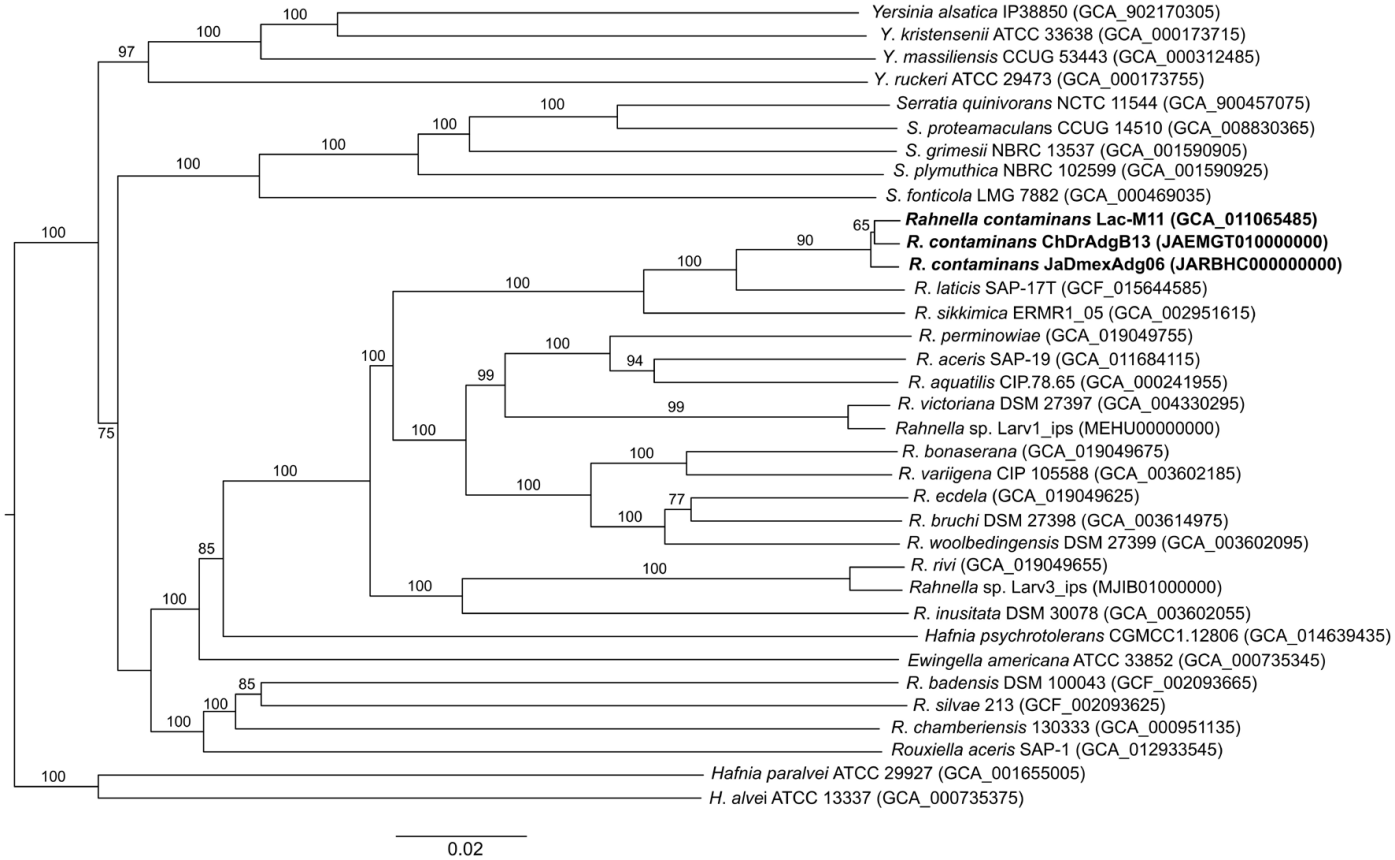
Phylogenetic tree of all *Rahnella* nominal species, including ChDrAdgB13 and JaDMexAdg06 strains. The tree was constructed using GBDP distances among genomes. Bootstrap values >57% are shown at the nodes.

The assembled genomes of strains ChDrAdgB13 and JaDmexAd06 were 5.74 Mb (5,732,748 bp) in length, consisting of 257 contigs with a N50 length of 0.58 Mbp, including only one copy of 16S rRNA, 54 tRNAs, 538 duplicate genes, and 26 functional categories based on the KEGG pathway database; for the second, of 5.54 Mb (5,544,148 bp) in length, 67 contigs with a N50 of 0.78 Mbp, one copy 16S rRNA, 70 tRNAs, 511 duplicate genes, and 26 functional categories. CheckM-estimated completeness for both genomes was 99.98% for ChDrAdgB13 and 94.54% for JaDmexAd06. The coverage depth of these strains was 104x for ChDrAdgB13 and 100x for JaDmexAd06. The genomic DNA G + C content for ChDrAdgB13 and JaDmexAd06 was similar to *R*. *contam inans* (53.1%) and other *Rahnella* species whose percentage varied from 51.9% (*R*. *bruchi* DSM 27398^T^) to 53.7% (*R*. *victoriana* DSM 27397^T^). The pairwise ANI values of the strains ChDrAdgB13 and JaDmexAd06, with respecto to *Rhanella* species, varied from 84.02–84.07% (*R*. *inusitada* DSM 30038^T^) to 99.13–99.18% (*R*. *contaminans* LacM11^T^)(Table 2), meanwhile dDDH values of both strains respect to *R*. *contaminans* was >93.0, but <60.0 (27.5–60.9) with respect other *Rahnella* species (Table 2). The phylogenomic tree (Figure 4) showed that both strains integrate, together with *R*. *contaminans*, a consistent and well-defined cluster (98% bootstrap value) within the *Rahnella* genus.

**Table 2:** Pairwise average nucleotide identity (ANI, bottom left) and digital DNA-DNA hybridization (d_4_DDH, top right) among ChDrAdgB13 and JaDmexAdg06 strains, and nominal *Rahnella* species.

ANI/dDDH	1	2	3	4	5	6	7	8	9	10	11	12	13	14	15	16	17	18
1	**–**	92.5	93.9	60.9	30.2	27.8	27.5	30.0	29.8	29.4	29.6	29.5	30.1	29.7	30.1	28.7	27.8	45.7
2	99.08	**–**	93.4	60.7	30.1	27.8	27.5	29.9	29.9	29.4	29.6	29.5	30.0	29.7	30.0	28.9	27.9	45.6
3	99.13	99.18	**–**	60.8	30.0	27.8	27.5	30.0	29.8	29.4	29.6	29.8	30.0	29.6	29.9	28.6	27.8	45.7
4	94.84	95.03	95.01	**–**	29.6	27.8	27.9	29.1	28.8	28.9	28.7	29.2	29.5	28.7	28.9	28.2	27.8	50.2
5	85.63	85.63	85.62	85.53	**–**	29.2	28.1	34.8	33.3	36.4	34.1	37.3	89.7	34.5	34.2	34.3	29.2	29.8
6	84.48	84.45	84.42	84.34	85.13	**–**	32.5	28.7	28.8	28.7	28.8	29	29.2	28.6	28.6	28.1	85.9	27.8
7	84.02	84.07	84.17	84.41	84.44	87.14	**–**	27.5	27.6	27.4	27.3	27.7	28.2	27.3	27.4	26.9	32.6	28.1
8	85.41	85.8	85.61	85.33	88.1	84.7	84.1	**–**	42.9	34.7	42.2	33.7	34.5	43.5	54.2	32.4	28.88	29.2
9	85.52	85.61	85.7	84.93	87.39	84.85	84.05	91.02	**–**	33.3	53	33.1	33.2	47.6	42.4	31.6	28.7	28.9
10	85.48	85.75	85.47	85.19	88.85	84.86	83.97	88.01	87.27	**–**	33	49.2	36.5	33.1	34.1	42.3	28.7	29.0
11	85.58	85.56	85.61	84.99	87.83	84.91	83.94	90.84	93.43	87.28	**–**	32.9	32.9	53.5	42.1	31.4	28.5	28.8
12	85.57	85.74	85.61	85.17	89.34	85.05	84.3	87.91	87.36	92.68	87.31	**–**	37.7	32.9	33.8	45.3	28.9	29.3
13	85.73	85.66	85.6	85.47	98.74	85.16	84.41	87.92	87.48	88.88	87.22	89.42	**–**	33.1	33.9	34.1	29.3	29.6
14	85.58	85.64	85.56	85.03	87.89	84.93	84.09	91.09	92.18	87.34	93.76	87.56	87.38	**–**	42.8	31.6	28.6	28.7
15	85.64	86.77	85.67	85.09	87.88	84.61	84.0	93.78	90.71	88.01	90.6	87.77	87.67	90.81	**–**	42.8	28.8	28.8
16	84.76	84.87	84.85	84.59	87.64	84.42	83.55	86.86	86.47	90.83	86.43	91.72	87.68	86.42	86.81	**–**	28.2	28.3
17	84.37	84.36	84.43	84.31	85.27	98.28	87.26	84.8	84.83	84.86	84.81	85.11	85.29	84.83	84.63	84.43	**–**	27.8
18	92.0	92.01	92.05	93.15	85.75	84.64	84.61	85.34	85.29	85.50	85.30	85.48	85.68	85.31	85.11	84.73	85.54	**–**

Strains: 1, *R. contaminans* ChDrAdgB13; 2, *R. contaminans* JaDmexAdg06; 3, *R. contaminans* (Lac M11^T^); 4, *R. laticis* (SAP-17T^T^); 5, *Rahnella* sp. Larv1_ips; 6, *Rahnella* sp. Larv3_ips; 7, *R. inusitata* (DSM 30078^T^); 8, *R. variigena* (CIP 105588^T^); 9, *R. woolbedingensis* (DSM 27399^T^); 10, *R. aceris* (SAP-19^T^); 11, *R. bruchi* (DSM 27398^T^); 12, *R. aquatilis* (LMG 2794^T^); 13, *R. victoriana* (DSM 27397^T^); 14, *R. ecdela* (DSM 112612^T^); 15, *R. bonaserana* (DSM 112610^T^); 16, *R. perminowiae* (DSM 112609^T^); 17, *R. rivi* (DSM 112611^T^); 18, *R. sikkimica* (ERMR1_05 ^T^).

**Figure 5:**
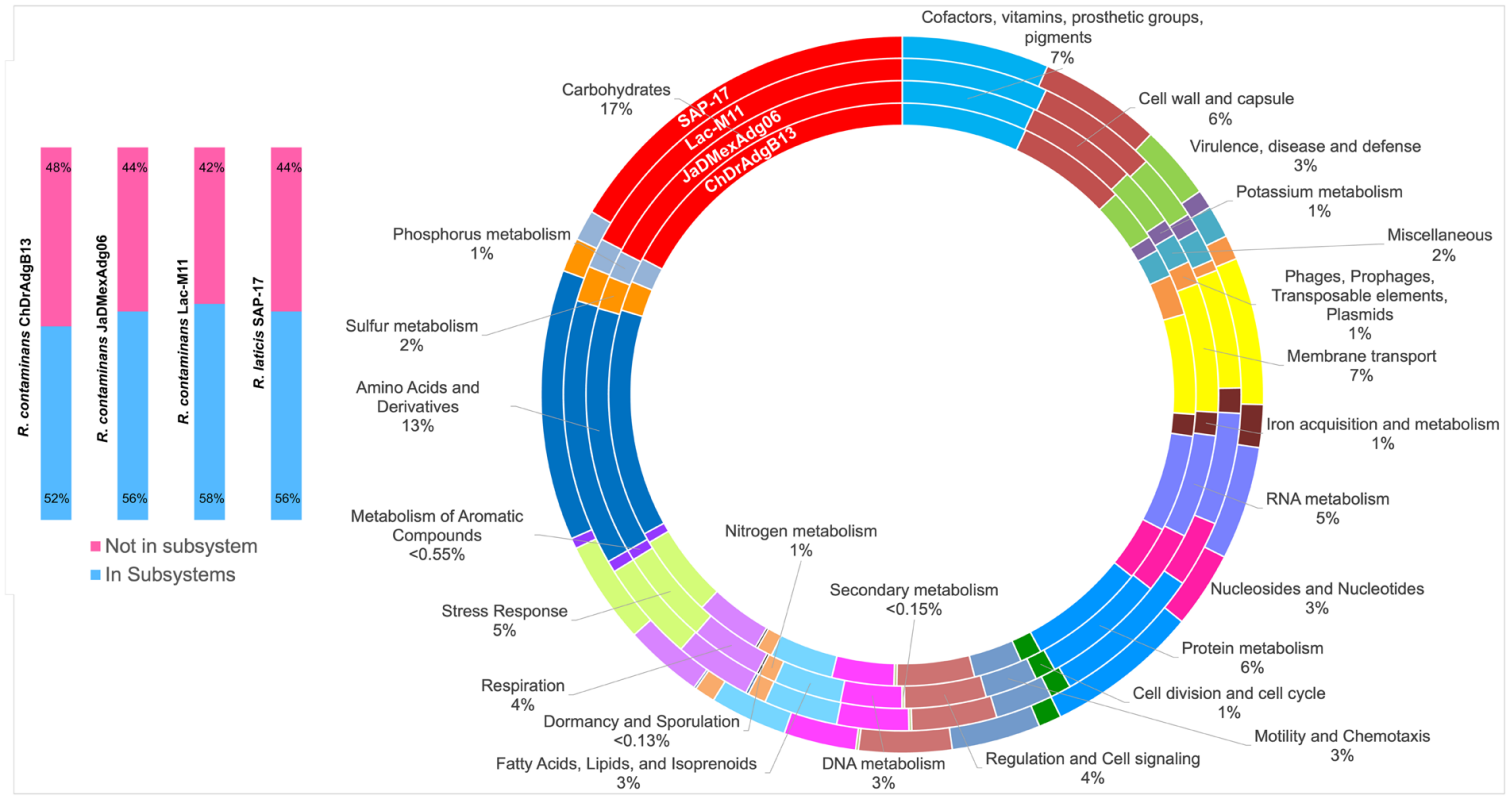
Genome features of *Rahnella contaminans* ecotype (ChDrAdgB13 and JaDMexAdg06 strains) and, type strains *R*. *contaminans* and *R*. *laticis*. The circles show the 26 functional categories annotated in Rapid Annotation System Technology Server. The blue color corresponds to the percentage of proteins associated with subsystems, and the pink to proteins not associated.

The overview of genomes of both strains showed genes associated with carbohydrate metabolism (17.29–17.47%), amino acids and derivatives (12.6–12.7%), cofactors, vitamins, prosthetic groups and pigments (6.8–6.9%), protein metabolism (6.38–6.46%), cell wall and capsule (5.75–6.05%), membrane transport (5.5–6.7%), RNA metabolism (5.3–5.37%), stress response (4.57–4.89%), and detoxification (4%). The genomes of *R*. *contaminans* and *R*. *laticis* show similar percentages to *R*. *contaminans* ecotype strains in these and other KEGG-functional categories, except for protein metabolism where a higher number of genes were found in both species (7.27% *R*. *contaminans* and 6.86% *R*. *laticis*), and a lower number of genes for carbohydrate degradation in *R*. *laticis* (16.48%) (Figure 5 and Supplementary Table S4).

In particular, the genomes of ChDrAdgB13 and JaDmexAd06 have genes involved in xylan hydrolysis (endo-1,4-β-xylanase, α-xylosidases, α-L-arabinofuranosidase and a partial sequence of one β-xylosidase) and other carbohydrates such as cellulose (β-glucosidases) and starch (cytoplasmic α-amylase, α-glucosidases, and β-glucosidases), which might contribute to the nutrition of host insects. These same genes were found in the genomes of *R*. *contaminans* and in *R*. *laticis*.

The *in silico* analysis of the flagellar system of the strains ChDrAdgB13, JaDmexAd06, and *Rahnella* species showed the presence of *flag*-1 primary system encoding for peritrichous flagella (Supplementary Table S5). This system is integrated by 50 genes encoding proteins involved in the biosynthesis, regulation, maintenance, and chemotaxis of this flagella type. The only exceptions are *R*. *variigena* (CIP 105588), which incorporates a second system, the *flag*-3, which also encodes for peritrichous flagella, and *R*. *laticis* (SAP-17), which presents the *fla*-A gene, a member of the *flag*-5 system encoding for amphitrichous (polar) flagella (Supplementary Table S5).

## Discussion

The genus *Rahnella* includes species capable of living in diverse aquatic and terrestrial habitats (Izard et al., 1979; Brady et al., 2014; Lee et al., 2020; Jeon et al., 2021; Brady et al., 2022), including extremophile environments (Kumar et al., 2022). In this study, a comprehensive attempt to clarify the taxonomic status of *Rahnella* isolates associated with the gut of *Dendroctonus*-bark beetles was carried out using phenotypic, molecular, and phylogenetic evidence.

Based on the recommended criteria in the International Code of Nomenclature of Bacteria for naming species, genomospecies, or subspecies (Parker et al., 2019), the differences observed between isolates and nominal species of *Rahnella* do not allow arguing in favor of some of these categories for our isolates. Yet, ANI and dDDH values and phylogenetic inference analyses with the 16S rRNA, MLSA, and genomes indicate that the gut-associated *Rahnella* of these beetles is an ecotype of *R*. *contaminans*.

*Rahnella contaminans* was isolated and described as a contaminant of refrigerated MRSA agar plates after the isolation of *Lactobacillus* strains using raw rice wine; however, re-isolation of strains of this species using the same isolation conditions was not possible (Jeon et al., 2021). By contrast, *R*. *contaminans* ecotype associated with *Dendroctonus* species has been recurrently isolated from the gut of these bark beetles; its persistence across the different development stages of these insects and their geographical localities (Hernández-García et al., 2017; Briones-Roblero et al., 2017a,b) indicates that it is a persist symbiont whose isolation source and habitat are common. The bark beetles’ gut is a distinctive habitat by its morphology, toxic environment, and physicochemical properties, which influence and determine, in combination with the interactions between microbes and between these and their host, the species that make up the microbial assemblage.

Considering that the sources of isolation were very different, it was amazing to find a great phenotypic similarity and close phylogenetic relationship between this ecotype and *R*. *contaminans*. Yet, various aspects must be highlighted. The first is that MLSA and phylogenomic analyses, either with whole genomes or with the core genome, recover the genus *Rahnella* as a monophyletic group independent of other genera of the family *Yersiniaceae*, where *R*. *contaminans* and the ecotype integrate a well-defined cluster (Figures 3, 4). This group was also recovered with 16S rRNA, despite low phylogenetic resolution (Supplementary Figure S1). Second, the flagellar system of ChDrAdgB13, JaDmexAd06 and all *Rahnella* species is *flag*-1, except in *R*. *variigena* which in addition to *flag*-1 also has *flag*-3; yet, both systems encode for peritrichous flagella (Supplementary Table S5). This result is in agreement with the type of flagellum observed in the ecotype by TEM and with observations made in *Rahnella* species in other studies, which report the same flagella type (Izard et al., 1979; Brady et al., 2014, 2022; Kumar et al., 2022). However, Lee et al. (2020) and Jeon et al. (2021) reported and showed images of amphitrichous flagella in *R*. *contaminans*, *R*. *aceris*, and *R. laticis*, suggesting that all *Rahnella* species have this type of flagella. This consideration does not agree with the flagellar loci present in the genome of *R*. *contaminans*, as well as other *Rahnella* species, or with the peritrichous flagella observed in the ecotype and *Rahnella* species. Finally, a noteworthy observation in this study is the presence of fimbriae in the ecotype (Figure 2), which could be an adaptation to the intestinal environment. Fimbriae are mainly involved in adhesion to different inerts or living surfaces, which favors colonization (Thanassi et al., 2007). In the gut, these structures could help bacteria to adhere to epithelial cells, which is essential for the colonization or invasion of host tissue. This explains the dominance and persistence of this ecotype along all developmental stages in bark beetles (Briones-Roblero et al., 2017a). Unfortunately, this feature has been overlooked in the description of *Rahnella* species, which does not allow us to know whether these structures are expressed by all species, regardless of growth conditions or habitat. Yet, an overview of the genome of *Rahnella* species showed that they have fimbriae genes from the family types 1, α, β, and σ mainly encoding chaperone/usher fimbriae, as well as other uncharacterized families (Thanassi et al., 2007; Supplementary Table S6).

In brief, this study shows that *Rahnella* isolates associated with the gut of *Dendroctonus*-bark beetles are an ecotype of *R. contaminans*. This ecotype is a recurrent, dominant, and persistent symbiont in the gut, which plays a fundamental ecological role in the benefit of these bark beetles, due to its capacity to hydrolyze esters, lipids, and xylans (Briones-Roblero et al., 2017b; Pineda-Mendoza et al., 2022), as well as recycle uric acid (Morales-Jiménez et al., 2013), and degrade or transform different toxic monoterpenes for insects (Boone et al., 2013; Xu et al., 2015, 2016).

### Emended description of the *Rahnella contaminans* (Jeon et al., 2021)

The following characteristics are added or modified in the description of *Rahnella contaminans* given by Jeon et al. (2021). All these features were obtained under toxic growth conditions according to Brady et al. (2014, 2022).

Cells were rods (0.4–0.6 × 2–3.5 μm), single or in pairs, motile by peritrichous flagella and fimbria. Colonies are white, round, smooth, convex with entire border, bright, and do not produce diffusible pigment after 48 h of growth at 28°C in TSA medium. Cultures grow well between 20 and 28°C and pH range from 4 to 10; optimal growth at 28°C and pH 7. Positive for β-galactosidase, tryptophan deaminase, and indole and acetoin production. Negative for arginine dihydrolase, lysine decarboxylase, ornithine decarboxylase, citrate utilization, H_2_S, and urease. Nitrate is reduced to nitrite. Acid is produced from L-arabinose, D-ribose, D-xylose, D-galactose, D-glucose, D-fructose, D-mannose, L-rhamnose, dulcitol, D-mannitol, N-acetylglucosamine, arbutin, esculin, ferric citrate, salicin, D-cellobiose, D-maltose, D-lactose, D-melibiose, D-saccharose, D-trehalose, D-raffinose, gentiobiose, and D-fucose (API 50CHB/E). Dextrin, glycogen, tweens 40 and 80, N-acetyl-D-glucosamine, L-arabinose, D-arabitol, D-cellobiose, D-fructose, L-fucose, D-galactose, gentiobiose, α-D-glucose, α-D-lactose, maltose, D-mannitol, D-mannose, D-melibiose, D-raffinose, L-rhamnose, D-sorbitol, D-trehalose, sucrose, turanose, pyruvic acid methyl ester, acetic acid, citric acid, formic acid, D-galactonic acid lactone, D-galacturonic acid, D-gluconic acid, malonic acid, quinic acid, D-saccharic acid, glucuronamide, L-alaninamide, D-alanine, L-alanine, L-alanylglycine, L-asparagine, L-aspartic acid, L-glutamic acid, glycyl-L-aspartic acid, glycyl-L-glutamic acid, D-serine, L-serine, inosine, uridine, thymidine, glycerol, D, L-α-glycerol phosphate, α-D-glucose-1-phosphate, and α-D-glucose-6-phosphate (Biolog) are oxidized. Reactions for cis-aconitic acid and bromosuccinic acid are negative. The main fatty acids include C_12:0_, C_14:0_, C_16:0_, C_17:0_ cyclo, iso-C_16:1_ and/or C_14:0_ 3-OH (summed feature 2) and C_16:1_ ω7*c* and/or C_16:1_ω6c (summed feature 3). The G + C content of the ecotype was 52.8 mol%. The ecotype of *R*. *contaminans* showed the presence of *flag*-1 primary system encoding peritrichous flagella, as well as fimbriae genes from the families type 1, α, β, and σ, mainly encoding chaperone/usher fimbriae. The ecotype of *R. contaminans* was recurrently isolated from the gut of *Dendroctonus*-bark beetles, where it is persistent across the different development stages of these insects and geographical localities.

## Data availability statement

The datasets presented in this study can be found in online repositories. The names of the repository/repositories and accession number(s) can be found in the article/Supplementary material.

## Author contributions

GZ and FR-O conceived the research. BV-C performed the experiment. XZ and WC performed the chemotaxonomic analysis. FR-O, RP-M, ML, CC-R, and GZ performed the *in silico* analysis, interpreted the result, and performed the draft and final edition of the manuscript. All authors contributed and approved the final manuscript.

## Funding

This research was partially funded by Consejo Nacional de Ciencia y Tecnología (CONACyT-Ciencia de la Frontera 1311330) and Secretaría de Investigación y Posgrado del IPN (20221751). This study was part of the BV-C MsC thesis. BV-C was fellow CONACYT (576556) and the Member of the Program of Beca de Estímulo Institutional de Formación de Investigadores del Instituto Politécnico Nacional (BEIFI-IPN).

## Conflict of interest

The authors declare that the research was conducted in the absence of any commercial or financial relationships that could be construed as a potential conflict of interest.

## Publisher’s note

All claims expressed in this article are solely those of the authors and do not necessarily represent those of their affiliated organizations, or those of the publisher, the editors and the reviewers. Any product that may be evaluated in this article, or claim that may be made by its manufacturer, is not guaranteed or endorsed by the publisher.

## Footnotes

^1^ https://lpsn.dsmz.de/genus/rahnella

^2^ www.ezbiocloud.net

^3^ http://atgc.lirmm.fr/phyml/

^4^ https://blast.ncbi.nlm.nih.gov/Blast.cgi

^5^ http://www.atgc-montpellier.fr/phyml/

^6^ https://rast.nmpdr.org/

^7^ https://narrative.kbase.us

^8^ http://exon.gatech.edu/GeneMark/gmhmm2_prok.cgi

^9^ http://www.genome.jp/kegg/

^10^ http://enve-omics.ce.gatech.edu/ani/index

^11^ http://ggdc.dsmz.de/ggdc.php

^12^ https://tygs.dsmz.de

^13^ http://www.atgc-montpellier.fr/fastme/

^14^ https://www.bv-brc.org
