# Supplementary material for: A polyphasic taxonomy analysis reveals the presence of an ecotype of Rahnella contaminans associated with the gut of Dendroctonus-bark beetles
Source: Front Microbiol. 2023 Apr 27;14:1171164. doi: 10.3389/fmicb.2023.1171164 (PMC10174453; doi:10.3389/fmicb.2023.1171164)
Supplement: Supplementary file 1 [file Data_Sheet_1.docx]

Supplementary Material

A polyphasic taxonomy analysis reveals the presence of an ecotype of *Rahnella contaminans* associated with the gut of *Dendroctonus*-bark beetles

**Flor N. Rivera-Orduña^1^, Rosa María Pineda-Mendoza^2^, Brenda Vega-Correa^1^, María Fernanda López^2^, Claudia Cano-Ramírez^2^, Xiao Xia Zhang^3^, Wen Feng Chen^4^ and Gerardo Zúñiga^2*^.**

*** Correspondence:** Gerardo Zúñiga [capotezu@hotmail.com](mailto:capotezu@hotmail.com)

# Supplementary Tables

**Table S1.** Phenotypic characteristics of ten isolates of *Rahnella contaminans* associated to the gut of *Dendroctonus*-bark beetles.

| **Characteristic** | **1** | **2** | **3** | **4** | **5** | **6** | **7** | **8** | **9** | **10** |
| --- | --- | --- | --- | --- | --- | --- | --- | --- | --- | --- |
| Vogues-Proskauer | + | + | + | + | + | + | + | + | + | + |
| Acid from |  |  |  |  |  |  |  |  |  |  |
| D-Arabinose | + | + | + | + | + | + | + | + | + | + |
| D-Saccharose | + | + | + | + | + | + | + | + | + | + |
| D-Fucose | + | + | + | + | + | + | + | + | + | + |
| D-Arabitol | + | + | + | + | + | + | + | + | + | + |
| Potassium gluconate | - | - | - | - | - | - | - | - | - | - |
| Potassium-5-ketogluconate | - | - | - | - | - | - | - | - | - | - |
| Utilization of |  |  |  |  |  |  |  |  |  |  |
| N-acetyl-d-galactosamine | + | - | - | - | + | - | - | + | + | - |
| D-Arabitol | - | - | + | - | + | - | - | - | + | + |
| D-Sorbitol | + | + | - | + | + | + | + | + | + | + |
| cis- Aconitic acid | + | - | - | - | + | + | - | + | - | + |
| D-Galactonic acid lactone | + | - | - | - | - | - | + | + | - | + |
| D-Glucuronic acid | + | - | - | + | - | - | + | + | + | + |
| α- Ketobutyric acid | + | - | - | - | - | - | + | + | - | + |
| α- Ketoglutaric acid | - | - | - | - | + | + | - | + | - | - |
| Succinamic acid | + | - | - | - | - | - | - | + | - | + |
| Urocanicacid | - | - | - | - | - | - | - | - | - | - |
| Enzyme activities |  |  |  |  |  |  |  |  |  |  |
| Arginine dihydrolase | **-** | - | - | - | - | - | - | - | - | - |
| β- galactosidase | **+** | + | + | + | + | + | + | + | + | + |
| Tryptophan deaminase | + | + | + | + | + | + | + | + | + | + |

**1.**ChDrLvgB08; **2.** ChDrAdgB13; **3.** ChDrPegB23; **4.** ChDrPugB47; **5.** JaDadAdg04, **6.** JaDadAdg19; **7**. JaDvAdg08; **8.** JaDvLvg39; **9.** JaDmexAdg06; **10.** JaDmexAdg07.

**Table S2.** Fatty acid profiles of ten isolates of *Rahnella contaminans* associated to the gut of *Dendroctonus*-bark beetles.

| **Fatty acid** | **1** | **2** | **3** | **4** | **5** | **6** | **7** | **8** | **9** | **10** |
| --- | --- | --- | --- | --- | --- | --- | --- | --- | --- | --- |
| C_12:0_ | 2.92 | 2.94 | 2.91 | 2.84 | 2.76 | 2.89 | 2.92 | 2.91 | 2.91 | 2.91 |
| C_12:0_ 3-OH | 0.10 | 0.09 | 0.09 | 0.07 | 0.11 | 0.09 | 0.10 | 0.08 | 0.12 | 0.09 |
| C_13:0_ | 0.06 | 0.08 | 0.05 | 0.08 | 0.06 | 0.09 | 0.07 | 0.07 | 0.06 | 0.09 |
| C_14:0_ | 5.51 | 5.57 | 5.60 | 5.55 | 5.50 | 5.57 | 5.48 | 5.61 | 5.67 | 5.50 |
| C_16:0_ | 26.42 | 26.55 | 26.54 | 26.58 | 26.50 | 26.53 | 26.65 | 26.49 | 26.50 | 26.61 |
| C_16:0_ ω5*c* | 0.08 | 0.11 | 0.12 | 0.10 | 0.11 | 0.13 | 0.09 | 0.14 | 0.13 | 0.15 |
| C_17:0_ | 0.39 | 0.37 | 0.33 | 0.35 | 0.39 | 0.34 | 0.40 | 0.33 | 0.35 | 0.40 |
| C_17:0_ ω7*c* | 0.16 | 0.17 | 0.19 | 0.15 | 0.19 | 0.14 | 0.15 | 0.18 | 0.12 | 0.18 |
| C_18:0_ | 0.41 | 0.40 | 0.43 | 0.40 | 0.45 | 0.44 | 0.48 | 0.43 | 0.41 | 0.38 |
| C_17:0_ cyclo | 22.73 | 22.71 | 22.82 | 22.67 | 22.76 | 22.75 | 22.70 | 22.82 | 22.77 | 22.69 |
| C_19:0_ cyclo ω8*c* | 0.89 | 0.84 | 0.80 | 0.88 | 0.84 | 0.79 | 0.82 | 0.89 | 0.88 | 0.80 |
| C_19:0_ iso | 0.05 | 0.08 | 0.10 | 0.07 | 0.12 | 0.06 | 0.09 | 0.08 | 0.11 | 0.07 |
| **Summed features** |  |  |  |  |  |  |  |  |  |  |
| **2**: iso-C_16:1_ and/or C_14:0_ 3-OH | 8.39 | 8.36 | 8.30 | 8.41 | 8.33 | 8.35 | 8.42 | 8.39 | 8.32 | 8.36 |
| **3**: C_16:1_ ω7*c* and /or C_16:1_ω6c | 15.40 | 15.44 | 15.42 | 15.48 | 15.41 | 15.49 | 15.40 | 15.37 | 15.35 | 15.42 |

**1.**ChDrLvgB08; **2.** ChDrAdgB13; **3.** ChDrPegB23; **4.** ChDrPugB47; **5.** JaDadAdg04, **6.** JaDadAdg19; **7**. JaDvAdg08; **8.** JaDvLvg39; **9.** JaDmexAdg06; **10.** JaDmexAdg07.

**Table S3.** The sequence similarity (%) of 16S rRNA gene (lower left) and concatenated housekeeping genes *gyrB, rpoB*, *infB* and *atpD* (MLSA) (upper right) of the ten strains of *R. contaminans* and nominal species of the genus *Rahnella*.

|  | **1** | **2** | **3** | **4** | **5** | **6** | **7** | **8** | **9** | **10** | **11** | **12** | **13** | **14** | **15** | **16** | **17** | **18** | **19** | **20** | **21** | **22** | **23** | **24** |
| --- | --- | --- | --- | --- | --- | --- | --- | --- | --- | --- | --- | --- | --- | --- | --- | --- | --- | --- | --- | --- | --- | --- | --- | --- |
| **1** |  | 99.02 | 99.38 | 99.02 | 99.47 | 99.14 | 99.26 | 99.38 | 99.30 | 99.14 | 99.51 | 97.02 | 92.30 | 95.56 | 95.39 | 95.31 | 95.52 | 94.21 | 94.74 | 95.51 | 94.82 | 93.60 | 92.87 | 93.72 |
| **2** | 99.70 | - | 99.38 | 99.59 | 98.98 | 99.38 | 99.51 | 99.38 | 99.51 | 98.98 | 99.67 | 96.57 | 92.05 | 95.39 | 95.47 | 95.35 | 95.15 | 94.17 | 94.74 | 95.35 | 94.94 | 93.64 | 92.54 | 93.52 |
| **3** | 99.70 | 99.40 | - | 99.47 | 99.34 | 99.51 | 99.63 | 99.83 | 99.67 | 99.10 | 99.51 | 96.74 | 92.26 | 95.39 | 95.60 | 95.19 | 95.31 | 94.33 | 94.86 | 95.27 | 95.03 | 93.76 | 92.74 | 93.60 |
| **4** | 99.70 | 99.70 | 99.70 | - | 99.14 | 99.30 | 99.59 | 99.47 | 99.51 | 99.06 | 99.34 | 96.65 | 92.17 | 95.31 | 95.39 | 95.23 | 95.07 | 94.25 | 94.70 | 95.19 | 94.86 | 93.64 | 92.62 | 93.52 |
| **5** | 99.62 | 99.33 | 99.92 | 99.62 | - | 99.02 | 99.38 | 99.42 | 99.34 | 99.42 | 99.34 | 96.82 | 92.42 | 95.64 | 95.35 | 95.31 | 95.48 | 94.09 | 94.62 | 95.43 | 94.78 | 93.56 | 92.87 | 93.64 |
| **6** | 99.92 | 99.77 | 99.62 | 99.92 | 99.55 | - | 99.63 | 99.42 | 99.34 | 99.10 | 99.63 | 96.65 | 92.05 | 95.23 | 95.43 | 97.10 | 95.23 | 94.17 | 94.70 | 95.19 | 94.90 | 93.60 | 92.54 | 93.43 |
| **7** | 99.92 | 99.77 | 99.62 | 99.92 | 99.55 | 99.70 | - | 99.71 | 99.63 | 99.38 | 99.06 | 96.78 | 92.26 | 95.43 | 95.56 | 97.26 | 95.27 | 94.29 | 94.82 | 95.31 | 95.03 | 93.72 | 92.70 | 93.60 |
| **8** | 99.77 | 99.47 | 99.47 | 99.77 | 99.40 | 99.70 | 99.70 | - | 99.67 | 99.18 | 99.42 | 96.74 | 92.26 | 95.47 | 95.60 | 97.22 | 95.31 | 94.33 | 94.86 | 95.35 | 95.03 | 93.76 | 92.70 | 93.56 |
| **9** | 99.33 | 99.18 | 99.03 | 99.33 | 98.95 | 99.40 | 99.40 | 99.25 | - | 99.18 | 99.10 | 96.57 | 92.09 | 95.39 | 95.51 | 97.30 | 95.15 | 94.25 | 94.78 | 95.19 | 94.94 | 93.68 | 92.58 | 93.52 |
| **10** | 99.70 | 99.77 | 99.40 | 99.70 | 99.33 | 99.77 | 99.77 | 99.47 | 99.18 | - | 99.18 | 96.66 | 92.14 | 95.23 | 95.11 | 97.88 | 95.23 | 93.85 | 94.38 | 95.23 | 94.58 | 93.28 | 92.58 | 93.52 |
| **11** | 99.92 | 99.77 | 99.62 | 99.92 | 99.55 | 99.77 | 99.77 | 99.70 | 99.40 | 99.77 | - | 99.34 | 93.18 | 93.87 | 95.64 | 97.95 | 93.77 | 93.04 | 93.04 | 93.34 | 92.88 | 92.71 | 93.53 | 93.90 |
| **12** | 98.58 | 98.28 | 98.88 | 98.58 | 98.81 | 98.50 | 98.50 | 98.42 | 98.14 | 98.28 | 98.51 | - | 92.30 | 94.74 | 94.25 | 97.96 | 94.74 | 93.77 | 94.17 | 94.62 | 93.89 | 93.40 | 93.24 | 94.25 |
| **13** | 99.18 | 99.03 | 99.03 | 99.18 | 98.95 | 99.10 | 99.10 | 98.80 | 98.73 | 99.03 | 99.25 | 97.98 | - | 92.75 | 92.06 | 97.10 | 93.07 | 91.81 | 91.61 | 92.78 | 91.44 | 91.20 | 94.91 | 93.43 |
| **14** | 98.77 | 98.44 | 98.44 | 98.77 | 98.36 | 98.69 | 98.69 | 98.77 | 98.20 | 98.44 | 98.69 | 98.93 | 98.28 | - | 95.92 | 97.80 | 98.16 | 93.81 | 94.99 | 97.31 | 94.74 | 93.28 | 92.58 | 92.82 |
| **15** | 98.73 | 98.43 | 98.43 | 98.73 | 98.36 | 98.65 | 98.65 | 98.57 | 98.14 | 98.43 | 98.66 | 98.95 | 98.43 | 99.75 | - | 99.06 | 95.43 | 95.31 | 95.76 | 96 | 96.94 | 94.66 | 91.85 | 92.45 |
| **16** | 98.28 | 97.99 | 97.99 | 98.28 | 97.91 | 98.20 | 98.20 | 98.05 | 97.69 | 97.99 | 98.21 | 97.39 | 98.51 | 98.12 | 98.14 | - | 97.96 | 98.94 | 98.90 | 97.96 | 98.98 | 98.98 | 96.94 | 98.21 |
| **17** | 98.73 | 98.43 | 98.43 | 98.73 | 98.36 | 98.65 | 98.65 | 98.57 | 98.14 | 98.43 | 98.66 | 98.88 | 98.43 | 99.75 | 99.92 | 98.21 | - | 93.89 | 94.78 | 96.90 | 94.46 | 93.36 | 92.75 | 92.82 |
| **18** | 97.76 | 97.47 | 98.06 | 97.76 | 97.99 | 97.68 | 97.68 | 97.53 | 97.17 | 97.47 | 97.69 | 97.54 | 98.13 | 97.63 | 97.69 | 99.47 | 97.76 | - | 96.00 | 94.09 | 95.72 | 96.82 | 91.28 | 91.88 |
| **19** | 98.58 | 98.28 | 98.88 | 98.58 | 98.81 | 98.50 | 98.50 | 98.42 | 97.99 | 98.28 | 98.51 | 99.18 | 98.36 | 99.50 | 99.47 | 97.91 | 99.47 | 98.06 | - | 94.90 | 95.76 | 95.27 | 91.24 | 92.20 |
| **20** | 98.73 | 98.43 | 98.43 | 98.73 | 98.36 | 98.65 | 98.65 | 98.57 | 98.14 | 98.43 | 98.66 | 98.88 | 98.43 | 99.75 | 99.92 | 98.21 | 99.47 | 97.76 | 99.47 | - | 95.23 | 93.35 | 92.70 | 92.66 |
| **21** | 98.28 | 98.14 | 98.58 | 98.28 | 98.51 | 98.35 | 98.35 | 98.13 | 97.84 | 98.14 | 98.36 | 99.10 | 98.13 | 99.26 | 99.25 | 97.69 | 99.18 | 97.84 | 99.47 | 99.18 | - | 95.19 | 91.49 | 91.96 |
| **22** | 98.43 | 98.14 | 98.14 | 98.43 | 98.06 | 98.35 | 98.35 | 98.20 | 97.76 | 98.14 | 98.36 | 97.62 | 98.06 | 98.36 | 98.36 | 99.25 | 98.43 | 98.88 | 98.14 | 98.43 | 97.91 | - | 91.16 | 91.55 |
| **23** | 98.36 | 98.21 | 98.36 | 98.36 | 98.28 | 98.42 | 98.42 | 98.20 | 97.91 | 98.21 | 98.43 | 98.73 | 98.28 | 99.34 | 99.33 | 97.69 | 99.25 | 97.54 | 99.40 | 99.25 | 99.47 | 97.91 | - | 93.52 |
| **24** | 98.81 | 98.51 | 98.66 | 98.81 | 98.58 | 98.72 | 98.72 | 98.65 | 98.36 | 98.51 | 98.73 | 99.55 | 98.06 | 99.10 | 99.10 | 97.62 | 99.03 | 97.32 | 98.88 | 99.03 | 98.81 | 97.84 | 98.58 | - |

Strains*:* 1 ChDrAdgB13, 2 JaDmexAdg06, 3 ChDrLvgB08, 4 ChDrPegB23,5. ChDrPugB47, 6 JaDadAdg04, 7 JaDadAdg19, 8 JaDvAdg08, 9 JaDvLvg39, 10 JaDmexAdg07, 11 *R*. *contaminans* (Lac M11^T^), 12 *R*. *laticis* (SAP-17T^T^), 13 *R. inusitata* (DSM 30078^T^), 14 *R. variigena* (CIP 105588^T^), 15 *R. woolbedingensis* (DSM 27399^T^), 16 *R*. *aceris* (SAP-19^T^), 17 *R. bruchi* (DSM 27398^T^), 18 *R*. *aquatilis* (LMG 2794^T^), 19 *R. victoriana* (DSM 27397^T^), 20 *R*. *ecdela* (DSM 112612^T^), 21 *R*. *bonaserana* (DSM 112610^T^), 22 *R*. *perminowiae* (DSM 112609^T^), 23 *R*. *rivi* (DSM 112611^T^), 24 *R*. *sikkimica* (ERMR1_05^T^)

**Table S4**. Genome features of the *Rahnella* *contaminans* ecotype (ChDrAdgB13 y JaDMexAdg06 strains) and the type strains from *R*. *contaminans* Lac-M11 and *R*. *laticis* SAP-17.

| Genome subsystems features | *R*. *contaminans*  ChDrAdgB13 | *R*. *contaminans*  JaDMexAdg06 | *R*. *contaminans* Lac-M11 | *R*. *laticis*  SAP-17 |
| --- | --- | --- | --- | --- |
| Sequence size (bp) | 5,732,748 | 5,544,148 | 5,230,797 | 5,727,497 |
| Number of contigs | 257 | 67 | 15 | 23 |
| GC content (%) | 52.8 | 52.9 | 53.1 | 52.9 |
| Shortest contig size | 1,001 | 532 | 1,079 | 2,405 |
| Median sequence size | 5,341 | 4,864 | 124,470 | 156,661 |
| Mean sequence size | 22,306.4 | 82,748 | 348,719.8 | 249,021 |
| Longest contig size | 242,954 | 2,076,605 | 2,062,847 | 719,657 |
| N50 value | 58,349 | 788,191 | 1,200,290 | 468,686 |
| L50 value | 27 | 2 | 2 | 5 |
| Number of subsystems | 555 | 550 | 546 | 565 |
| Number of coding sequences | 5,410 | 5,037 | 4,746 | 5,180 |
| Number of RNAs | 54 | 70 | 79 | 71 |
| Cofactors, vitamins, prosthetic groups, pigments | 267 | 263 | 265 | 263 |
| Cell wall and capsule | 234 | 221 | 221 | 219 |
| Virulence, disease and defense | 114 | 116 | 121 | 133 |
| Potassium metabolism | 33 | 33 | 33 | 33 |
| Miscellaneous | 60 | 60 | 60 | 59 |
| Phages, Prophages, Transposable elements, Plasmids | 87 | 46 | 20 | 44 |
| Membrane transport | 214 | 256 | 217 | 265 |
| Iron acquisition and metabolism | 47 | 46 | 46 | 77 |
| RNA metabolism | 208 | 205 | 204 | 204 |
| Nucleosides and Nucleotides | 118 | 122 | 122 | 137 |
| Protein metabolism | 250 | 245 | 276 | 274 |
| Cell division and cell cycle | 46 | 40 | 38 | 41 |
| Motility and Chemotaxis | 103 | 103 | 104 | 159 |
| Regulation and Cell signaling | 165 | 160 | 154 | 166 |
| Secondary metabolism | 6 | 5 | 5 | 5 |
| DNA metabolism | 132 | 120 | 130 | 129 |
| Fatty Acids, Lipids, and Isoprenoids | 135 | 133 | 134 | 138 |
| Nitrogen metabolism | 33 | 34 | 33 | 37 |
| Dormancy and Sporulation | 5 | 5 | 5 | 4 |
| Respiration | 140 | 141 | 140 | 141 |
| Stress Response | 177 | 188 | 184 | 185 |
| Metabolism of Aromatic Compounds | 21 | 21 | 21 | 19 |
| Amino Acids and Derivatives | 488 | 488 | 485 | 487 |
| Sulfur metabolism | 64 | 65 | 65 | 61 |
| Phosphorus metabolism | 53 | 54 | 54 | 55 |
| Carbohydrates | 669 | 671 | 659 | 658 |

**Table S5**. *In silico* analysis of the flagellar system of the *R. contaminans* ecotype (ChDrAdgB13 and JaDmexAd06 strains) and nominal species of the genus *Rahnella.*

| **Protein** | **Strains** | | | | | | | | | | | | | | | | | |
| --- | --- | --- | --- | --- | --- | --- | --- | --- | --- | --- | --- | --- | --- | --- | --- | --- | --- | --- |
|  | **1** | **2** | **3** | **4** | **5** | **6** | **7** | **8** | **9** | **10** | **11** | **12** | **13** | **14** | **15** | **16** | **17** | **18** |
| Flagellar basal-body P-ring formation protein FlgA | + | + | + | + (2) | + | + | + | + (2) | + | + | + (2) | + | + | + | + | + | + | 1 |
| Flagellar basal-body rod protein FlgB | + | + | + | + (2) | + | + (2) | + | + (2) | + | + | + (2) | + | + | + | + | + | + | + |
| Flagellar basal-body rod protein FlgC | + | + | + | + (2) | + | + | + | + (2) | + | + | + (2) | + | + | + | + | + | + | + |
| Flagellar basal-body rod modification protein FlgD | + | + | + | + (2) | + | + | + | + (2) | + | + | + (2) | + | + | + | + | + | + | + |
| Flagellar hook protein FlgE | + | + | + | + (2) | + | + | + | + (2) | + | + | + (2) | + | + | + | + | + | + | + |
| Flagellar basal-body rod protein FlgF | + | + | + | + | + | + | + | + | + | + | + | + | + | + | + | + | + | + |
| Flagellar basal-body rod protein FlgG | + | + | + | + (2) | + | + (2) | + | + (2) | + | + | + (2) | + | + | + | + (2) | + | + | + |
| Flagellar L-ring protein FlgH | + | + | + | + | + | + (2) | + | + (2) | + | + (2) | + (2) | + | + | + | + (2) | + | + | + |
| Flagellar P-ring protein FlgI | + | + | + | + (2) | + | + (2) | + | + (2) | + | + | + (2) | + | + | + | + (2) | + | + | + |
| Flagellar protein FlgJ [peptidoglycan hydrolase] | + | + | + | + | + | + | + | + (2) | + | + | + (2) | + | + | + | + | + | + | + |
| Flagellar hook-associated protein FlgK | + | + | + | + (2) | + | + | + | + (2) | + | + | + (2) | + | + | + | + | + | + | + |
| Flagellar hook-associated protein FlgL | + | + | + | + (2) | + | + | + | + (2) | + | + | + (2) | + | + | + | + | + | + | + |
| Flagellar biosynthesis protein FlgN | + | + | + | + | + | + | + | + (2) | + | + | + | + | + | + | + | + | + | + |
| Negative regulator of flagellin synthesis FlgM | + | + | + | + | + | + | + | + | + | + | + | + | + | + | + | + | + | + |
| Flagellar protein FlhE | + | + | + | + | + | + | + | + | + | + | + | + | + | + | + | + | + | + |
| Flagellar biosynthesis protein FlhA | + | + | + | + (2) | + | + (2) | + | + (2) | + | + | + (2) | + | + | + | + (2) | + | + | + |
| Flagellar biosynthesis protein FlhB | + | + | + | + (2) | + | + | + | + (2) | + | + | + (2) | + | + | + | + | + | + | + |
| Flagellar motor rotation protein MotA | + | + | + | + (2) | + | + (2) | + | + (2) | + | + | + (2) | + | + | + | + (2) | + | + | + |
| Flagellar motor rotation protein MotB | + | + | + | + (2) | + | + | + | + (2) | + | + | + (2) | + | + | + | + | + | + | + |
| Flagellar transcriptional activator FlhC | + | + | + | + | + | + | + | + (2) | + | + | + (2) | + | + | + | + | + | + | + |
| Flagellar transcriptional activator FlhD | + | + | + | + | + | + | + | + (2) | + | + | + (2) | + | + | + | + | + | + | + |
| Chemotaxis response - phosphatase CheZ | + | + | + | + (2) | + | + (2) | + | + (2) | + | + | + (2) | + | + | + | + (2) | + | + | + |
| Chemotaxis regulator - transmits chemoreceptor signals to flagellar motor components CheY | + | + | + (2) | + (2) | + | + | + | + (2) | + | + | + (2) | + | + | + | + (2) | + | + | + |
| Chemotaxis response regulator protein-glutamate methylesterase CheB | + | + | + | + (2) | + | + (2) | + | + (2) | + | + | + (2) | + | + | + | + (2) | + | + | + |
| Chemotaxis protein methyltransferase CheR | + | + | + | + (2) | + | + (2) | + | + (2) | + | + | + (2) | + | + | + | + (2) | + | + | + |
| Methyl-accepting chemotaxis protein I (mcp l) | + | + | + | + | + | + | + | + | + | + | + | + | + | + | + | + | + | + |
| Methyl-accepting chemotaxis citrate transducer (mcp) | + | + | + | + | + | + | + | + | + | + | + | + | + | + | + | + | + | + |
| Positive regulator of CheA protein activity (CheW) | + | + | + | + (2) | + | + (2) | + | + (2) | + | + | + (2) | + | + | + | + (2) | + | + | + |
| Signal transduction histidine kinase CheA | + | + | + | + (2) | + | + (2) | + | + (2) | + | + | + (3) | + | + | + (2) | + (2) | + | + | + |
| Chemotaxis protein CheV | + | + | + | + | + | + | + | + | + | + | + | + | + | + | + | + | + | + |
| Regulator of sigma S factor FliZ | + | + | + | + | + | + | + | + | + | + | + | + | + | + | + | + | + | + |
| RNA polymerase sigma factor for flagellar operon FliA | + | + | + | + | + | + | + | + | + | + | + | + | + | + | + | + | + | + (2) |
| Flagellin FliC | + | + | + | + | + | + (2) | + | + (2) | + | + | + (2) | + | + | + | + (6) | + | + | + |
| Flagellar cap protein FliD | + | + | + | + (2) | + | + | + | + (2) | + | + | + (2) | + | + | + | + | + | + | + |
| Flagellar biosynthesis protein FliS | + | + | + | + (2) | + | + | + | + (2) | + | + | + (2) | + | + | + | + | + | + | + |
| Flagellar biosynthesis protein FliT | + | + | + | + | + | + | + | + | + | + | + | + | + | + | + | + | + | + |
| Flagellar hook-basal body complex protein FliE | + | + | + | + (2) | + | + | + | + (2) | + | + | + (2) | + | + | + | + | + | + | + |
| Flagellar M-ring protein FliF | + | + | + | + (2) | + | + (2) | + | + (2) | + | + | + (2) | + | + | + | + (2) | + | + | + |
| Flagellar motor switch protein FliG | + | + | + | + (2) | + | + (2) | + | + (2) | + | + | + (2) | + | + | + | + (2) | + | + | + |
| Flagellar assembly protein FliH | + | + | + | + (2) | + | + | + | + (2) | + | + | + (2) | + | + | + | + | + | + | + |
| Flagellum-specific ATP synthase FliI | + | + | + | + (2) | + | + (2) | + | + (2) | + | + | + (2) | + | + | + | + | + | + | + |
| Flagellar protein FliJ | + | + | + | + | + | + | + | + (2) | + | + | + (2) | + | + | + | + | + | + | + |
| Flagellar hook-length control protein FliK | + | + | + | + (2) | + | + | + | + (2) | + | + | + (2) | + | + | + | + | + | + | + |
| Flagellar basal body-associated protein FliL | + | + | + | + | + | + | + | + (2) | + | + | + (2) | + | + | + | + | + | + | + |
| Flagellar motor switch protein FliM | + | + | + | + (2) | + | + (2) | + | + (2) | + | + | + (2) | + | + | + | + (2) | + | + | + |
| Flagellar motor switch protein FliN | + | + | + | + (2) | + | + (2) | + | + (2) | + | + | + (2) | + | + | + | + (2) | + | + | + |
| Flagellar biosynthesis protein FliO | + | + | + | + | + | + | + | + | + | + | + | + | + | + | + | + | + | + |
| Flagellar biosynthesis protein FliP | + | + | + | + (2) | + | + (2) | + | + (2) | + | + | + (2) | + | + | + | + | + | + | + |
| Flagellar biosynthesis protein FliQ | + | + | + | + (2) | + | + | + | + (2) | + | + | + (2) | + | + | + | + | + | + | + |
| Flagellar biosynthesis protein FliR | + | + | + | + (2) | + | + | + | + (2) | + | + | + (2) | + | + | + | + | + | + | + |
|  |  |  |  |  |  |  |  |  |  |  |  |  |  |  |  |  |  |  |
| Flagellar brake protein YcgR | + | + | + | + | + | + | + | + | + | + | + | + | + | + | + | + | + | + |
| Flagellar regulator flk | + | + | + | + | + | + | + | + | + | + | + | + | + | + | + | + | + | + |
| RNA polymerase sigma-54 factor RpoN | + (2) | + (2) | + (2) | + (2) | + (2) | + (2) | + (2) | + (2) | + (2) | + (2) | + (2) | + (2) | + | + | + (2) | + (2) | + (2) | + (2) |
| Flagellin protein FlaA (*flag*-5) | - | - | - | + | - | - | - | - | - | - | - | - | - | - | - | - | - | - |

Strains*:*1 *R*. *contaminans* ChDrAdgB13, 2 *R*. *contaminans* JaDmexAdg06, 3 *R*. *contaminans* (Lac M11^T^), 4 *R*. *laticis* (SAP-17T^T^), 5 *Rahnella* sp. Larv1_ips, 6 *Rahnella* sp. Larv3_ips, 7 *R. inusitata*(DSM 30078^T^), 8 *R. variigena*(CIP 105588^T^), 9*R. woolbedingensis*(DSM 27399^T^), 10*R*. *aceris* (SAP-19^T^), 11*R. bruchi*(DSM 27398^T^), 12*R*.*aquatilis*(LMG 2794^T^), 13*R. victoriana*(DSM 27397^T^), 14 *R*. *ecdela* (DSM 112612^T^), 15 *R*. *bonaserana* (DSM 112610^T^), 16 *R*. *perminowiae* (DSM 112609^T^), 17 *R*. *rivi* (DSM 112611^T^), 18 *R*. *sikkimica* (ERMR1_05).

**Table S6.** Proteins involved in fimbriae biosynthesis of the *R*. *contaminans* ecotype (ChDrAdgB13 and JaDmexAd06 strains) and nominal species of the genus *Rahnella*.

| **Protein** | **Strains** | | | | | | | | | | | | | | | | | |
| --- | --- | --- | --- | --- | --- | --- | --- | --- | --- | --- | --- | --- | --- | --- | --- | --- | --- | --- |
|  | **1** | **2** | **3** | **4** | **5** | **6** | **7** | **8** | **9** | **10** | **11** | **12** | **13** | **14** | **15** | **16** | **17** | **18** |
| Type 1 fimbriae regulatory protein, FimB | +(2) | +(2) | +(2) | **+** | +(2) | **-** | **-** | +(2) | **-** | +(2) | **-** | **-** | +(2) | **-** | +(2) | **-** | **-** | **+** |
| Type 1 fimbriae regulatory protein, FimA | +(2) | **-** | **-** | +(4) | +(2) | **-** | **-** | **-** | +(2) | **-** | + | **-** | +(2) | +(2) | **-** | **-** | **-** | **+** |
| Chaperona protein fimC precursor | **+** | **-** | **-** | **+** | +(2) | **-** | **-** | **-** | **+** | **-** | +(2) | **-** | **+** | +(2) | **-** | **-** | **-** | + |
| Type 1 fimbriae anchoring protein FimD | +(6) | +(2) | +(3) | +(5) | +(6) | +(2) | **+** | +(8) | +(4) | +(5) | +(3) | +(3) | +(6) | +(5) | +(7) | +(3) | +(2) | +(2) |
| Type 1 fimbriae adaptor subunit, FimF | **-** | **-** | **-** | **-** | **-** | **-** | **-** | **-** | **+** | **-** | +(2) | **-** | **-** | +(4) | **-** | **-** | **-** | **-** |
| Type 1 fimbriae adaptor subunit, FimG | **-** | +(2) | +(4) | +(4) | **-** | +(2) | **-** | **+** | **-** | **-** | **-** | **-** | **-** | **+** | **+** | **-** | **-** | **+** |
| Mannose-specif adhesin FimH | **-** | **-** | **-** | **-** | **-** | **-** | **-** | **+** | **-** | **-** | **-** | **-** | **-** | **-** | **-** | **-** | **-** | **-** |
| Type 1 fimbriae regulatory protein, FimB/FimE | +(2) | +(2) | +(2) | +(3) | +(2) | **-** | **-** | +(2) | **-** | +(3) | **-** | **-** | +(2) | **-** | +(2) | **-** | **-** | +(2) |
| Type-1 frimbrial protein, A chain precursor | + | + | + | + | +(3) | - | + | + | +(2) | + | - | + | + | + | + | - | - | + |
| Alpha-fimbriae major subunit | + | + | + | +(2) | + | - | + | + | - | + | - | + | + | + | + | + | - | + |
| Alpha-fimbriae tip adhesin | +(2) | + | + | + | +(2) | - | + | +(2) | - | +(2) | - | +(2) | +(2) | +(2) | + | +(2) | - | + |
| Alpha-fimbriae usher protein | + | + | + | +(2) | +(2) | - | + | + | - | + | - | +(2) | + | + | + | + | - | + |
| Alpha-fimbriae chaperone protein | +(2) | + | + | +(2) | +(2) | - | + | +(2) | - | +(2) | - | +(2) | +(2) | + | +(2) | +(2) | - | - |
| beta-frimbriae probable major subunit | - | - | - | - | - | - | - | - | - | - | +(2) | - | - | - | + | - | - | - |
| beta-frimbriae usher protein | - | - | - | + | - | - | - | - | - | - | + | - | - | - | - | - | - | - |
| beta-frimbriae chaperone protein | - | - | - | - | - | - | - | - | - | - | + | - | - | - | + | - | - | - |
| Sigma-fimbriae chaperone protein | + | + | + | + | + | + | + | + | + | + | + | - | + | + | + | + | + | + |
| Sigma-fimbriae tip adhesin | + | + | + | + | + | + | + | + | + | +(2) | + | - | + | + | + | + | + | + |
| Sigma-fimbriae uncharacterized paralogous subunit | +(2) | +(2) | +(3) | +(2) | +(2) | +(2) | +(2) | +(2) | +(2) | +(2) | +(2) | - | +(2) | +(2) | +(2) | +(2) | +(2) | +(2) |
| Sigma-fimbriae usher protein | + | + | + | + | + | + | +(2) | + | + | + | + | - | + | + | + | + | + | + |
| Uncharacterized fimbrial chaperone YbgP | + | + | + | + | +(2) | + | + | + | + | + | +(2) | + | + | +(2) | + | + | + | + |
| Uncharacterized fimbrial-like protein YadN | +(2) | - | - | - | + | - | - | + | - | + | - | + | +(2) | - | - | - | - | - |
| Uncharacterized fimbrial-like protein YadK | + | - | - | - | + | - | - | + | - | + | - | + | + | - | - | - | - | - |
| Uncharacterized fimbrial-like protein YadL | + | - | - | - | + | - | - | + | - | + | - | + | + | - | - | - | - | - |
| Uncharacterized frimbrial-like protein YraH | - | - | - | - | - | - | - | - | - | - | - | - | - | - | + | - | - | - |
| Fimbrial adhesin EcpD | + | - | - | - | + | - | - | + | - | + | - | + | + | - | - | - | - | - |
| Fimbrial protein | +(3) | - | +(2) | + | + | + | - | +(2) | - | + | - | + | +(3) | - | - | + | +(2) | + |
| Fimbrial protein precursor | +(3) | + | + | +(2) | +(2) | +(2) | + | + | +(2) | + | +(2) | + | +(3) | +(2) | +(2) | + | + | +(2) |
| Frimbrial protein YadM-like | + | - | - | - | + | - | - | + | - | + | - | + | + | - | + | - | - | - |
| Fimbrial adhesin precursor | +(2) | - | - | - | + | - | - | +(2) | - | + | - | - | +(2) | +(2) | + | - | - | - |
| Fimbrial biogenesis outer membrane usher protein | + | - | - | - | - | - | - | + | - | - | - | - | + | - | - | - | - | - |
| Fimbriae usher protein StfC | - | - | - | + | - | - | - | + | - | - | - | - | - | - | - | - | + | - |
| Fimbrial protein YadC | + | + | + | - | - | - | - | - | - | - | - | + | + | - | - | - | - | - |
| Frimbrial subunit BcfE | - | - | - | - | - | - | - | - | - | - | - | - | - | + | - | - | - | - |
| Frimbrial chaporene BcFB | - | - | - | - | - | - | - | - | - | - | - | - | - | + | - | - | - | - |
| Putative fimbrial-like protein | + | - | + | - | + | - | - | + | + | + | - | + | + | + | + | + | - | - |
| Putative outer membrane usher protein YqiG | +(7) | + | + | + | +(4) | + | + | + | + | + | +(2) | + | +(7) | +(2) | + | + | + | + |
| Putative fimbrial chaparone | +(2) | + | + | +(2) | +(2) | + | - | +(2) | + | + | +(3) | +(2) | +(2) | + | +(3) | + | +(2) | +(2) |
| Putative fimbrial subunit | - | - | + | - | - | - | - | - | - | - | - | + | - | - | - | - | - | - |
| Periplasmic fimbrial chaperone StfD | - | - | + | - | - | - | - | - | - | - | - | - | - | - | - | - | - | - |
| FIG031703: Fimbriae usher protein StbC | + | - | - | + | +(2) | + | + | + | - | - | - | - | + | - | - | + | + | - |
| FIG085450: Frimbrial protein | + | +(2) | + | - | +(2) | + | + | +(2) | - | + | + | + | + | + | + | + | + | +(2) |
| CFA/I Frimbrial subunit C usher protein | - | - | - | - | - | - | - | - | - | - | + | - | - | - | +(2) | - | - | - |

Strains: 1 *R*. *contaminans* ChDrAdgB13, 2 *R*. *contaminans* JaDmexAdg06, 3 *R*. *contaminans* (Lac M11^T^), 4 *R*. *laticis* (SAP-17^T^), 5 *Rahnella* sp. Larv1_ips, 6 *Rahnella* sp. Larv3_ips, 7 *R*. *inusitata* (DSM 30078^T^), 8 *R*. *variigena* (CIP 105588^T^), 9 *R*. *woolbedingensis* (DSM 27399^T^), 10 *R*. *aceris* (SAP-19^T^), 11 *R*. *bruchi* (DSM 27398^T^), 12 *R*. *aquatilis* (LMG 2794^T^), 13 *R*. *victoriana* (DSM 27397^T^), 14 *R*. *ecdela* (DSM 112612^T^), 15 *R*. *bonaserana* (DSM 112610^T^), 16 *R*. *perminowiae* (DSM 112609^T^), 17 *R*. *rivi* (DSM 112611^T^),18 *R*. *sikkimica* (ERMR1_05^T^).

## Supplementary Figures


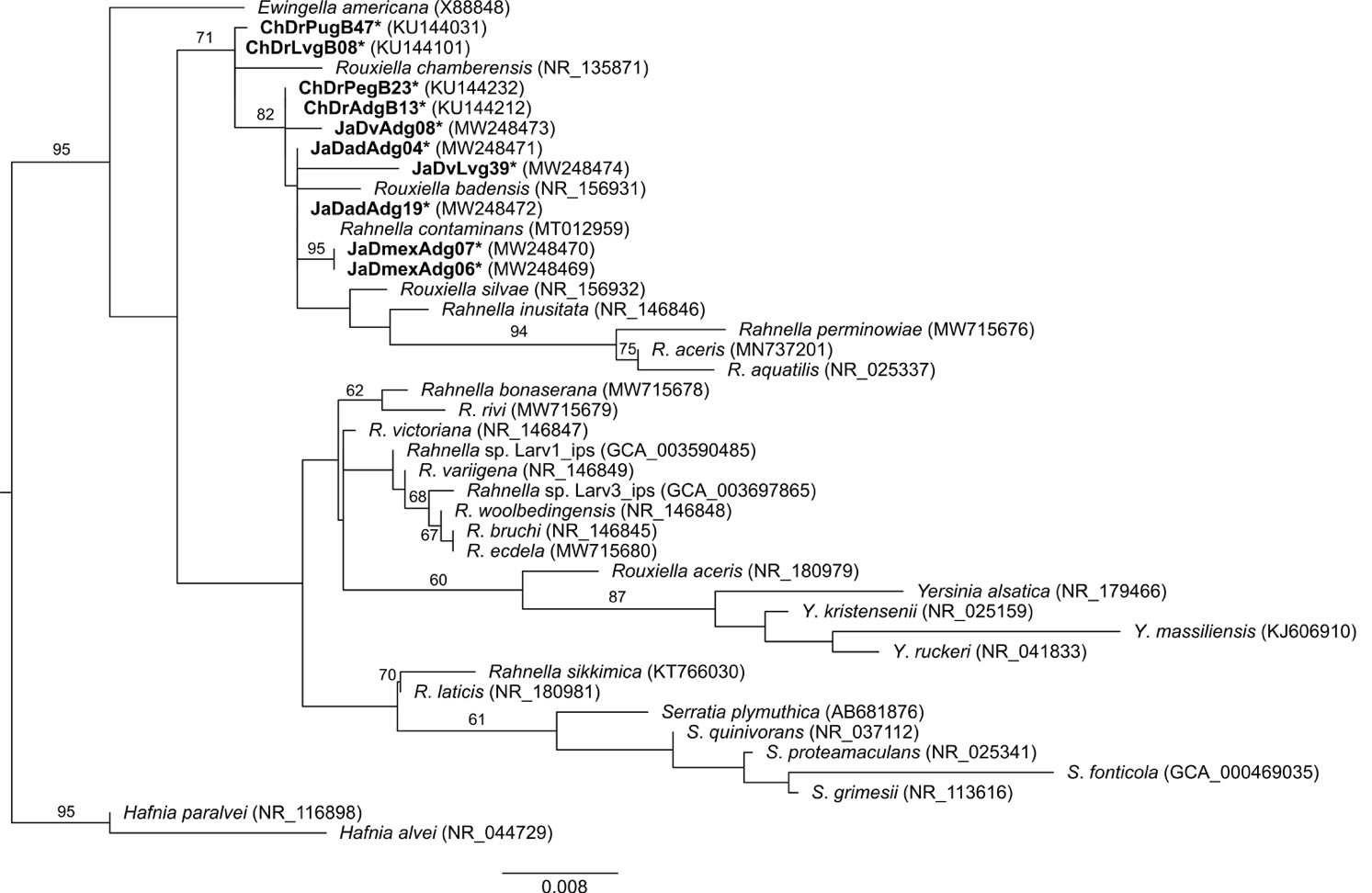


**Supplementary Figure 1.** Maximum likelihood phylogeny of the 16S rRNA sequences of ten isolates of *Rahnella contaminans* ecotype (*) and the type strains of the genus *Rahnella.* The nucleotide substitution model was HKY85+G+I with a gamma parameter of 0.592 according Akaike criterion. Bootstrap values >60% after 1000 pseudoreplications are shown at nodes.
